# Successful left atrial access through GORE CARDIOFORM ASD occluder using an integrated transseptal wire system after failed RF needle approach

**DOI:** 10.1002/joa3.70123

**Published:** 2025-06-30

**Authors:** Yuko Suzuki, Keijiro Nakamura, Takayuki Shimizu, Masako Asami, Hidehiko Hara

**Affiliations:** ^1^ Division of Cardiovascular Medicine Toho University Ohashi Medical Center Tokyo Japan

**Keywords:** atrial septal defect, cardiac electrophysiology, catheter ablation, structural heart disease, transseptal puncture

## Abstract

An integrated transseptal wire system enabled successful left atrial access through the elastic resistance posed by a large GORE CARDIOFORM ASD occluder after failed RF needle attempt, allowing large‐bore cryoballoon sheath advancement via sequential sheath technique for atrial fibrillation ablation without procedural complications.

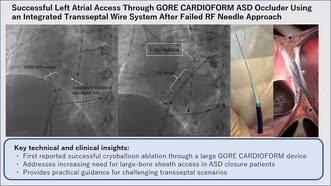

## 1 INTRODUCTION

The prevalence of atrial fibrillation (AF) in patients with atrial septal defects (ASD) is higher than in the general population, with rates exceeding 9% in the 40–50‐year age group.^1^, ^2^ Even after successful ASD closure, the annual incidence of new‐onset AF remains significant at approximately 4.1%,^2^ making subsequent left atrial interventions relatively common in this patient population. Transseptal puncture in patients with previous ASD occluder implantation presents distinct technical challenges. While studies suggest that transseptal access can be safely performed in select cases through occluder devices after complete endothelialization (typically 6 months postimplantation),^3^, ^4^ the specific techniques required vary depending on device characteristics. For occluders exceeding 26–28 mm in diameter, direct device puncture is often necessary because of insufficient native septal tissue.^3^

## 2 DESCRIPTION

### 2.1 Patient background

A 55‐year‐old male with a history of percutaneous ASD closure using a 44‐mm GORE® CARDIOFORM ASD Occluder (W. L. Gore & Associates) 2 years prior later developed paroxysmal AF requiring pulmonary vein isolation. Following ASD closure, minimizing the size of the transseptal access site is generally preferred, we were selecting a cryoballoon ablation was selected because of younger age, paroxysmal AF, and obstructive sleep apnea.

### 2.2 Technical considerations

Three key technical challenges were identified prior to the procedure: First, the large diameter (44‐mm) ASD occluder would necessitate direct device puncture because of insufficient native septal tissue. Second, appropriate puncture equipment selection was critical given the elastic properties of the GORE occluder material. Third, successful advancement of a 12‐Fr sheath would be required for cryoballoon therapy.

### 2.3 Initial Transseptal approach

**FIGURE 1:**
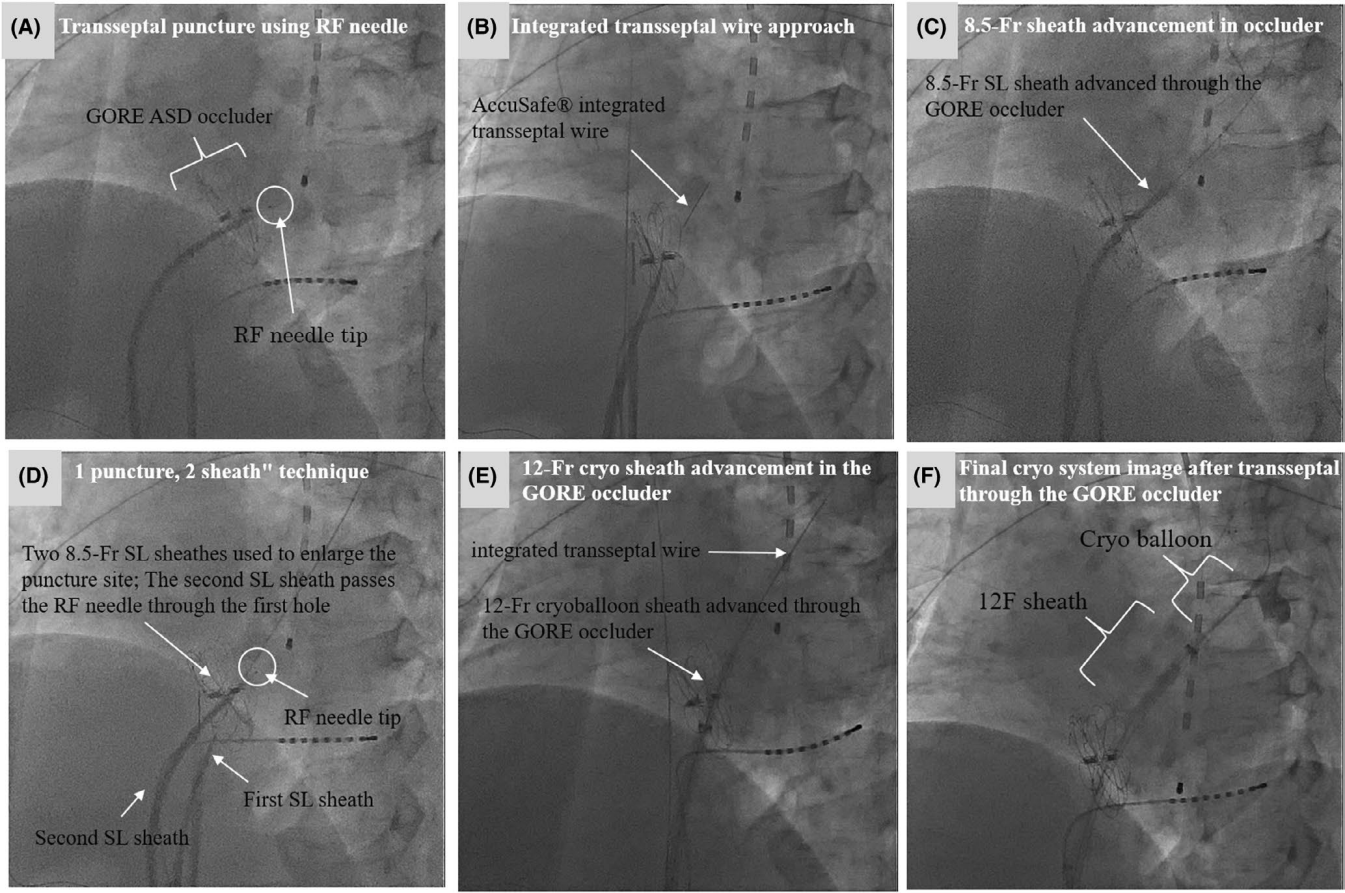
Sequential approach to transseptal access through GORE ASD occluder (LAO 55° view). (A) Transseptal puncture using RF needle: Initial transseptal puncture attempt using radiofrequency (RF) needle. Although the RF needle tip successfully penetrated the ASD occluder device, the outer sheath could not be advanced beyond the occluder material. (B) Integrated transseptal wire approach. Introduction of an integrated transseptal wire (AccuSafe® system) following unsuccessful sheath advancement with the RF needle. The integrated transseptal wire passes through the ASD occluder device. (C) 8.5‐Fr sheath advancement in occluder. Advancement of an 8.5‐Fr SL0 sheath through the occluder using the integrated wire system. (D) 1 puncture, 2 sheath” technique. “1 puncture, 2 sheath” technique: Sequential advancement of 8.5‐Fr SL0 sheath to enlarge the puncture site through the GORE occluder. (E) 12‐Fr cryosheath advancement in the GORE occluder. Successful advancement of a 12‐Fr cryoballoon sheath through the GORE occluder. (F) Final cryosystem image after transseptal through the GORE occluder. Final positioning of the cryoballoon system in the left atrium after transseptal access through the occluder.

During the procedure, a radiofrequency (RF) needle (Boston Scientific, Marlborough, MA, USA) was used in an attempt to puncture the GORE CARDIOFORM ASD occluder. Multiple radiofrequency energy applications (three times) were delivered to penetrate the occluder material (Figure 1A). Following successful penetration, the inner dilator advanced into the left atrium; however, the outer sheath could not be advanced through the device despite several attempts, likely because of the high elasticity and mechanical resistance of the ePTFE‐covered occluder (Figure 1B).

### 2.4 Alternative approach with integrated wire system

Subsequently, an AccuSafe® integrated transseptal puncture wire (Synaptic Medical Corporation) was employed (Figure 1C). This specialized tool features a needle with an integrated stiff guidewire that automatically forms a J‐shape after transseptal penetration. The J‐shaped configuration was confirmed fluoroscopically after puncture (Figure 1D).

### 2.5 Successful sheath advancement

Utilizing the integrated system's inherent stiffness, an 8.5‐Fr SL0 sheath was first introduced to enlarge the puncture site (Figure 1E), followed by the successful advancement of the 12‐Fr cryoballoon sheath (FlexCath Advance steerable sheath, Medtronic Inc.) without requiring balloon dilation (Figure 1F). No procedural complications occurred.

## 3 DISCUSSION

The GORE CARDIOFORM ASD occluder consists of a nitinol wire frame with a platinum core covered by expanded polytetrafluoroethylene (ePTFE). Unlike wire mesh‐based occluders such as the Amplatzer Septal Occluder (ASO), this construction presents specific challenges. Despite expectations that its structure would facilitate easier penetration because of reduced metal content, the high elasticity and mechanical strength of the ePTFE material created significant resistance during sheath advancement. The tensile strength of ePTFE exceeds that of polyester, explaining the increased difficulty in advancing sheaths through GORE occluders despite their seemingly favorable design.^5^

**FIGURE 2:**
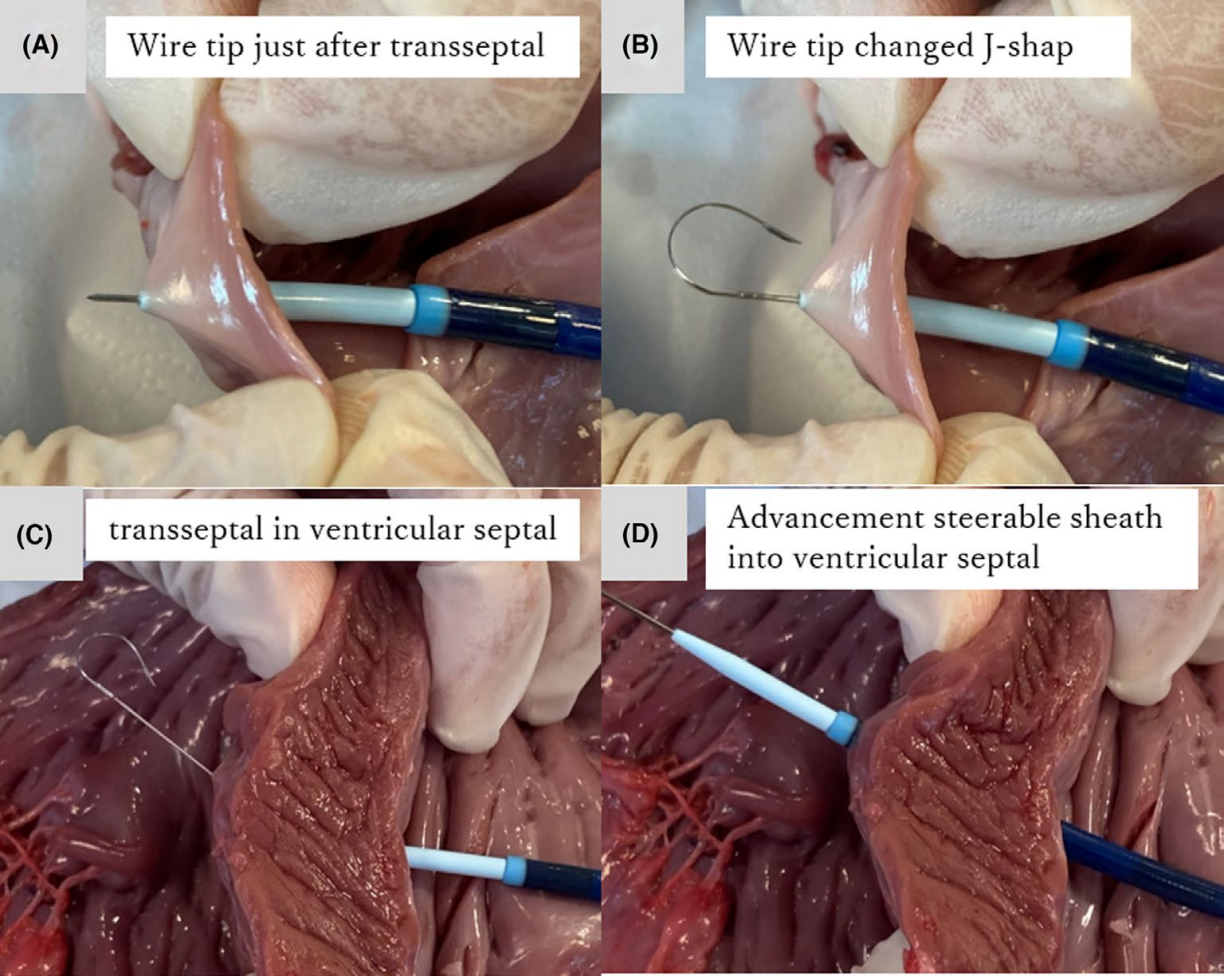
AccuSafe® integrated transseptal puncture wire system demonstration in porcine model. (A) Initial tenting of the atrial septum with an 8.5‐Fr sheath; the wire has just begun to cross the septum but remains in a straight configuration. (B) J‐shaped transformation of the wire tip after successful penetration of the atrial septum. (C) Advancement of the wire alone through the ventricular septum, showing full J‐tip formation. (D) Advancement of both the wire and the sheath through the ventricular septum.

The AccuSafe® integrated transseptal system offered several advantages in this challenging case: enhanced safety through the automatic J‐configuration after puncture, improved support via the stiff shaft enabling direct insertion of large‐bore sheaths without additional dilation steps, and elimination of wire exchange steps. Although these features were helpful in our case, it is important to note that clinical data specifically supporting the use of the AccuSafe® system remain limited. A recent case report described the use of the AccuSafe® wire in a patient with an atrial septal aneurysm, suggesting its possible usefulness in select anatomically difficult cases.^6^ Further studies are warranted to evaluate its safety, efficacy, and reproducibility in broader patient populations. To supplement our case findings, Figure 2 demonstrates the AccuSafe® system in a porcine model. The images show J‐tip formation after atrial septal puncture and successful wire and sheath advancement through the ventricular septum, highlighting the device's penetrability and shaft stiffness—even through thick myocardial tissue.

Our review of 10 cases of transseptal puncture after ASD closure at our institution revealed varying challenges based on device type (Table 1). While cases requiring specialized puncture tools had longer procedure times, all were ultimately successful. As shown in Table 1, our case (Case 7) was notable for being the only one involving a large GORE CARDIOFORM ASD occluder (44 mm) requiring cryoablation access. Other cases predominantly involved different occluder types that were generally smaller in size. Importantly, no cases of device damage have been reported in the literature, likely because of the elastic and compliant nature of modern occluders.^3^

**TABLE 1:** Characteristics of patients undergoing transseptal puncture through ASD closure devices.

	Gender	Age	Occluder type (size)	Time since ASD closure (months)	Transseptal puncture device	Ablation method	Complications
Case 1	F	62	ASO (unknown mm)	16	BRK	RF	None
Case 2	M	63	ASO (25 mm)	16	BRK	RF	None
Case 3	F	61	FSO (24 mm)	18	BRK	RF	None
Case 4	M	59	ASO (18 mm)	92	BRK	RF	None
Case 5	M	70	FSO (27 mm)	22	BRK	RF	None
Case 6	F	53	GCA (27 mm)	27	RF needle	RF	None
Case 7	M	55	GCA (44 mm)	31	ITWS after failed RF needle	Cryo	None
Case 8	M	79	FSO (30 mm)	46	BRK	RF	None
Case 9	M	64	FSO (30 mm)	62	ITWS after failed BRK	RF	None
Case 10	F	84	FSO (30 mm)	17	ITWS after failed RF needle	RF	None

Abbreviations: ASO, amplatzer septal occluder; BRK, Brockenbrough needle; Cryo, cryoablation; FSO, figulla septal occluder (Occlutech); GCA, GORE CARDIOFORM ASD occluder; ITWS, integrated transseptal puncture wire; RF, radiofrequency.

Compared to wire mesh‐based occluders such as the ASO, the GORE CARDIOFORM device—with its ePTFE covering, nitinol wire frame, and elastic properties—offers greater conformability but may generate more resistance during sheath advancement. Multicenter analyses and registries have indicated that transseptal access through GORE occluders can present greater technical challenges, with a higher frequency of alternative puncture techniques or specialized tools required.^5^, ^7^

This case highlights the technical considerations involved in transseptal access through a GORE CARDIOFORM ASD occluder and demonstrates how the use of specialized, wire‐integrated puncture tools can facilitate large‐bore sheath delivery in anatomically challenging situations.

### 3.1 Clinical implication

Wire‐integrated transseptal systems combine strong penetration capability with high shaft stiffness, enabling precise advancement through ASD occluders without loss of directional control within the right atrium. When paired with the “1 puncture, 2 sheath” technique, they may allow for the safe delivery of large‐bore sheaths. While RF ablation remains the standard approach in ASD closure case because of its simplicity and smaller sheath size, this technique could be considered in selected cases where cryoballoon or pulsed field ablation offers clinical benefit.

## FUNDING INFORMATION

None to declare.

## CONFLICT OF INTEREST STATEMENT

Authors declare no conflict of interests for this article.

## Supporting information


Data S1.
