# Supplementary figures and images for: Successful left atrial access through GORE CARDIOFORM ASD occluder using an integrated transseptal wire system after failed RF needle approach
Source: J Arrhythm. 2025 Jun 30;41(4):e70123. doi: 10.1002/joa3.70123 (PMC12207242; doi:10.1002/joa3.70123)

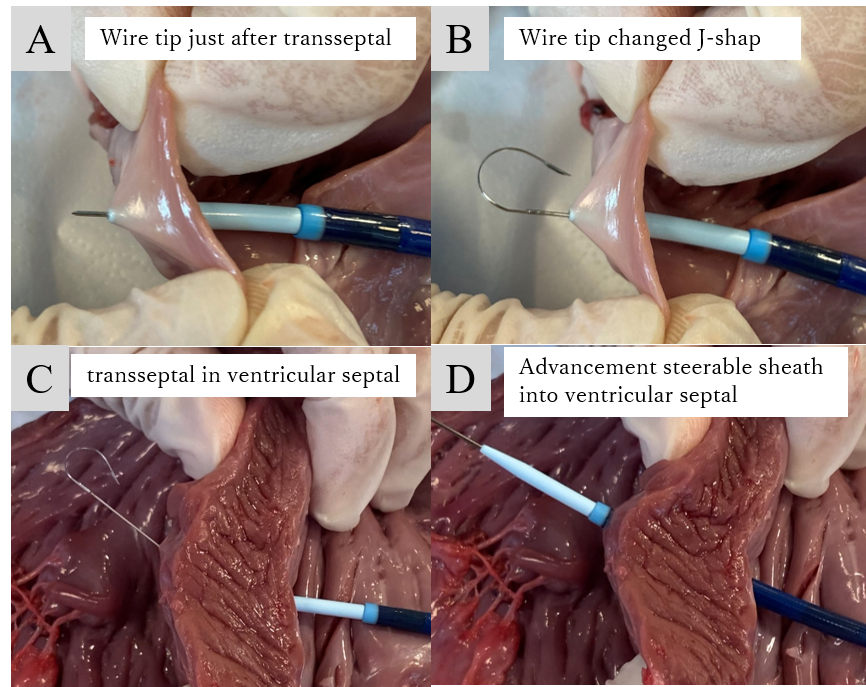

Supplement: Supplementary file 1 — Data S1. [file JOA3-41-e70123-s001.tif]
